# Diversity of fungal community and quality evaluation of *Spatholobus Suberectus* Dunn during the process of mildew

**DOI:** 10.1186/s13568-024-01665-9

**Published:** 2024-01-22

**Authors:** Chunfeng Xia, Yuchao Zhao, Chunlan Liu, Yang Gao

**Affiliations:** 1 College of Chemical and Biological Engineering, Yichun University, 336000 Yichun City, Jiangxi Province China; 2 College of Life Sciences and Resources and Environment, Yichun University, 336000 Yichun City, Jiangxi Province China

**Keywords:** *Spatholobus Suberectus* Dunn, Mildew, Fungal diversity, Flavonoids, Aflatoxin B_1_

## Abstract

*Spatholobus suberectus* Dunn as a traditional Chinese herbal medicine, which is susceptible to being infected by molds during storage. In order to explore the diversity characteristics of fungal community and the quality evaluation of *Spatholobus suberectus* Dunn during the process of mildew. The study used high-throughput sequencing technology to detect the diversity characteristics of fungal community, high-performance liquid chromatography (HPLC) and ultraviolet spectrophotometry (UV-spectrophotometry) methods to detect the content of flavonoids, and enzyme-linked immunosorbent assay (ELISA) method to detect the content of Aflatoxins B_1_ (AFB_1_). The result showed that the fungi of all samples belonged to 14 phyla, 336 genera, and the dominant fungi at the early stage of mildew was not obvious, while that at middle and late stages of mildew was *Aspergillus*. The species diversity of fungal community was the highest at the early stage of mildew, while the species richness of fungal community was the highest at the late stage of mildew. The content of AFB_1_ showed an upward trend, while the content of flavonoids showed a downward trend during the process of mildew. In brief, the diversity of fungal community decreased gradually, and the number of dominant fungi increased gradually, and the quality of *Spatholobus suberectus* Dunn decreased gradually during the process of mildew.

## Key points

**1st key point**: Explore the dominant fungi and the quality evaluation of *Spatholobus suberectus* Dunn during the process of mildew.

**2nd key point**: Dominant fungi are *Aspergillus* and the quality of *Spatholobus suberectus* decreased gradually.

**3rd key point**: Provide the theoretical basis for the development of new anti-molding technology.

## Introduction

Most of Chinese herbal medicine contain polysaccharides, starches, volatile oils and other components, which provide sufficient nutritional condition for growth of mold. Thus, when environment temperature is 25 ~ 35℃ and relative air humidity is over 75% or water content of medicinal materials is over 15%, mold can germinate hypha and produce an enzyme that can dissolve tissue and decompose active components of medicinal materials, causing medicinal value decline or even loss (Qi et al. 2023). In addition, some molds can produce mycotoxins, followed by the mycotoxin contamination (Zhao et al. 2017). Aflatoxins (AFs) were produced by *Aspergillus* strains, showing severe toxicity to liver and kidney, and high incidence in foods and medicinal materials. Nowadays, there are more than 20 types of AFs molecules and their isolated derivatives known. Among them, Aflatoxins B_1_ (AFB_1_) has strong hepatotoxic, and is classified as a Group IA carcinogen by the International Agency for Research on Cancer (IARC) (Liu et al. 2021; Duarte et al. 2020).

*Spatholobus suberectus* Dunn as a traditional Chinese herbal medicine is mainly distributed in Guangdong Province, Guangxi Province, Fujian Province and other southern regions of China (Zheng et al. 2012). It has effects of activating blood circulation, enriching blood, regulating menstruation, relieving pain, relaxing tendons and activating collaterals (Qin et al. 2018). *S. suberectus* Dunn has been reported that contains flavonoids, phenols, sterols, thraquinones, and terpenoids (Zhang and Xuan 2006; Tang et al. 2012; Yoon et al. 2004). Among them, flavonoids are the main active component, which has anti-inflammatory and antioxidant effects (Li et al. 2003; Chen et al. 2017). Hence, flavonoids are often used as the index component for quality evaluation of the *S. suberectus* Dunn decoction pieces (Huang et al. 2013).

At present, there are few reports about the relation between the diversity characteristics of fungal community and the quality of *S. suberectus* Dunn decoction pieces during the mildew process. Therefore, in this paper, diversity of fungal community by using high-throughput sequencing technology, the content of flavonoids and AFB_1_ as indexes for quality evaluation of *S. suberectus* Dunn decoction pieces, which was detected by using HPLC, UV-spectrophotometry and ELISA methods. The aim of the paper is to elucidate the diversity characteristics of fungal community and content variation trend of flavonoids and AFB_1_ in the *S. suberectus* Dunn decoction pieces during the process of mildew, which will provide the theoretical basis for the development of new anti-molding technology for Chinese herbal medicine.

## Materials and methods

### Preparation of samples

*S. suberectus* Dunn decoction pieces (batch number: 210,801, origin: Guangxi) was purchased from Chinese herbal medicine market of Zhangshu City, Jiangxi Province. Decoction pieces with reddish brown, large number of catheter holes and similar size were selected as samples. The samples were divided into three equal groups and each group was parallel three times. All samples were placed in an environment with a temperature of 28℃ and relative air humidity of 95%. The samples at first day were taken as samples at the early stage of mildew (A), those at fifth day were taken as samples at the middle stage of mildew (B), those at ninth day was taken as samples at the late stage of mildew (C). The samples were dipped into sterile water and shaken at speed of 2,000 rmp for 1.5 h, then enriching fungi of the extract with 0.22-µm detachable filter, the filters were collected and stored at -80℃ for further use. The remaining samples of each group were pulverized into powder and screened by 40 mesh, then stored at -80℃ for further use.

### Extraction of genomic DNA from samples

Fungi were collected from detachable filters, DNA of samples was extracted with DNA kits, and the purity and concentration of DNA were determined by 1% agarose gel electrophoresis.

### ITS library construction and high-throughput sequencing

Using the DNA of the sample as a template, ITS1F: 5’-CTTGGTCATTTAGAGGAAGTAA-3’ (5 µmol/L) and ITS2R: 5’-GCTGCGTTCTTCATCGATGC-3’ (5 µmol/L) were used for PCR amplification of the ITS region of samples. The PCR amplification volume was 20 µl: 5×FastPfu Buffer, 4 µl; dNTPs (2.5 mmol/L), 2 µl; Forward Primer (5 µmol/L), 0.8 µl; Reverse Primer (5 µmol/L), 0.8 µl; FastPfu Polymerase, 0.4 µg; BSA, 0.2 µl; DNA, 10 ng; Fill with double steaming water to 20 µl; The sequence parameters of PCR reaction were 95 °C and 3 min. Qualified purified samples were analysis by high-throughput sequencing with platform of Illumina NovaSeq6000.

### Sequencing data processing and statistical analysis

At first, the Trimmomatic v0.33 software (Bolger et al. 2014) was used to filter Raw Reads, and the Cutadapt 1.9.1 software (Martin 2011) was used to identify and remove primer sequences to obtain Clean Reads, which did not contain primer sequences. Finally, the dada2 method (Callahan et al. 2016) in the QIIME 2020.6 software (Bolyen et al. 2019) was used for denoising, double-ended sequences were spliced and chimeric sequences were removed to obtain Non-chimeric Reads. The Uparse software (Segata et al. 2011) was used to cluster Non-chimeric reads, and Non-chimeric reads were grouped into operational taxonomic units (OTUs) with 97% identity by default. Unite (Kõljalg et al. 2013) as a reference database, OTUs were annotated by using a Naive Bayes classifier, and then the community composition of each sample was counted at the phylum and genus level. The Shannon diversity index dilution curve was used to analyze the sequencing depth, and α-diversity, species composition, Principal ordinates analysis (PCoA) and Non-MetricMulti-Dimensional Scaling (NMDS) were used to evaluate the diversity structure of fungal community in the *S. suberectus* Dunn decoction pieces during the process of mildew. Determination of aflatoxin B_1_ content.

### Determination of aflatoxin B_1_ content

5.0 g of sample powder was accurately weighed and mixed with 25.0 ml of 70% methanol for oscillating extraction at 200 rpm for 10 min, proper amount of extract was transfer into a centrifuge tube, centrifuge at 5,000 rmp for 10 min, and the middle layer of supernatant was extract. 0.1 ml of extract mixed with 9.0 ml of sample diluent solution to obtain the test solution (dilution coefficient K = 50). The measured liquid will be detected by ELISA method. According to the regression equation of the AFB_1_ standard curve (1):

1 $$Y=-0.3017X+0.7245 ({R}^{2}=0.9229)$$

the corresponding logarithmic value lgC of concentration (C) can be obtained, then calculated its antilog to obtain the AFB_1_ concentration (C) of test solution, the content of AFB_1_ in the sample (W) can be calculated according to the Eq. (2):

2 $$W=C\times K$$

*W.* AFB_1_ content of samples (µg/Kg).

*C.* AFB_1_ content of test solution (µg/Kg).

*K.* Dilution coefficient of test solution.

### Determination of total flavonoids content

Referring to the extraction method of total flavonoids from *S. suberectus* Dunn by Zheng Jiexuan (Zheng et al. 2016). The 0.3 g of sample powder was mixed with 25.0 ml of 50% ethanol solution, which was extracted by using ultrasonic oscillation at 300 W, 45 Hz and 50℃ for 1.5 h, then the solution was filtered and centrifuged at 8,000 rmp for 10 min, the supernatant was the extract. The content of total flavonoids of extract was detected by aluminum nitrate colorimetric method (Lee et al. 2011). 1.0 ml of the extract was placed into 25.0 ml volumetric flask, mixed with 1.0 ml of 5% sodium nitrite, shaken well and left for 6 min, mixed with 1.0 ml of 10% aluminum nitrate, shaken well and left for 6 min, mixed with 10.0 ml of 1.0 mol/L sodium hydroxide solution, filled with 50% ethanol to the mark, then shaken well and left for 15 min. A_510nm_ were measured by UV-spectrophotometer, and the concentration of total flavonoids in the samples were calculated according to the regression equation of rutin standard curve (3):

3 $$Y=13.175X+0.0012 ({R}^{2} =0.9985)$$

### Determination of protocatechuic acid, catechin and epicatechin content

#### Preparation and content detection of extract

Referring to the method of detecting flavonoids content in the *S. suberectus* Dunn by Lu et al. (2018) 1.0 g of sample powder was accurately weighed and mixed with 10.0 ml of 80% methanol. Ultrasonic extraction was conducted at a frequency of 40 Hz and power output of 500 W for one hour at 50℃, followed by filtration. The filtrate was collected and centrifuged with speed of 12,000 rmp for 10 min at 4℃. The extract was obtained by diluting the supernatant 10 times and filtered with 0.22-µm organic membranes.

### Drawing of standard curves

The standard products of 4.9 mg of catechin, epicatechin and protocatechuic acid were weighed and dissolved with a small amount of 80% methanol respectively, then transferred into 5.0 ml volumetric flasks, filled with 80% methanol to produce 0.98 mg/ml standard stock solutions. The stock solution of each standard was accurately measured and diluted with 80% methanol to prepare seven different concentrations of mixed standard solutions, of which the mass concentrations of catechin standards were 196, 98, 49, 24.5, 12.25, 6.125, 3.0625 µg/ml, the mass concentrations of epicatechin standards were 392, 196, 98, 49, 24. 5, 12.25, 6.125 µg/ml, and the mass concentrations of protocatechuic acid standards were 196, 98, 49, 24.5, 12.25, 6.125, 3.0625 µg/ml. The mixed standard solution was filtered through 0.22-µm filter and then detected according to the chromatographic conditions in 2.7.3. The standard curves were drawn with the concentration (µg/ml) as the horizontal coordinate and the peak area as the vertical coordinate, and the regression equations were calculated respectively as follows: Eq. (4) represents catechin; Eq. (5) represent epicatechin, Eq. (6) represent protocatechuic acid:

4 $$Y=9814.6X-8090.3 ({R}^{2}=0.999)$$

5 $$Y=10985X-2632.8 ({R}^{2}=0.999)$$

6 $$Y=22458X-12,893 ({R}^{2}=0.999)$$

### Chromatographic conditions

Columns: Phenomenex Kinetex C18 (250 mm × 4.6 mm, 5 μm); Mobile phase: acetonitrile (A), 0.1% formic acid solution (B); Gradient elution (0 ~ 5 min, 9% ~ 11% A; 5 ~ 10 min, 11% ~ 12% A; 10 ~ 30 min, 12% ~ 16% A; 30 ~ 39 min, 16% A; 39 ~ 50 min, 16% ~ 19% A); Detection wavelength: 278 nm; Flow rate: 0.7 ml/min; Column temperature: 25 ℃; Sample size: 10 µl.

## Results

### Sequencing depth analysis

**Fig. 1:**
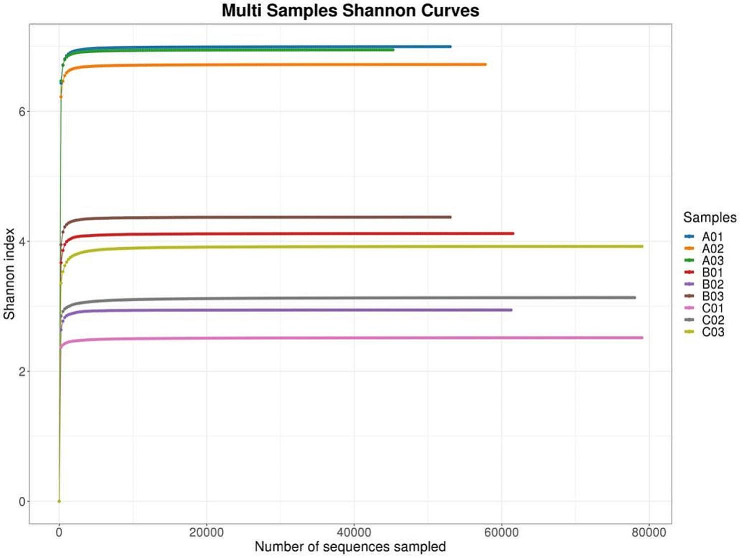
Shannon diversity index curves in samples from different groups of *Spatholobus suberectus* Dunn

The dilution curve of Shannon diversity index reflects the microbial diversity of each sample at different sequencing quantities. The larger the Shannon index, the greater the number of species, indicating that the majority of microbial species information is covered in the sample. When the curve tends to be flat, it means that the amount of sequencing data is large enough and the characteristic species will not increase with increasing sequencing volume. As shown in Fig. 1, the dilution curve of the sequencing sample tends to flat when the sequencing depth is 10,000, which indicated that the sequencing has become saturate. Hence, it can be considered that the sequencing depth has covered all species in the *S. suberectus* Dunn. samples and the amount of sequence data is sufficient to reflect the species diversity of *S. suberectus* Dunn samples.

### Statistical analysis of sequence data and fungal community structure composition

After the sequence optimization treatment, the 3 groups of samples with different mildew periods included early stage of mildew (A), middle stage of mildew (B) and late stage of mildew (C). The average number of Non-chimeric Reads in each group was 52,157 ± 6,355, 58,713 ± 4,834, 78,816 ± 693. By combining Non-chimeric Reads, and the average number of OTU in each group was 177 ± 19, 160 ± 25, 150 ± 131. Then the relative species abundance at the phylum and genus level of fungi after taxonomic annotation of OTUs in each group.

**Fig. 2:**
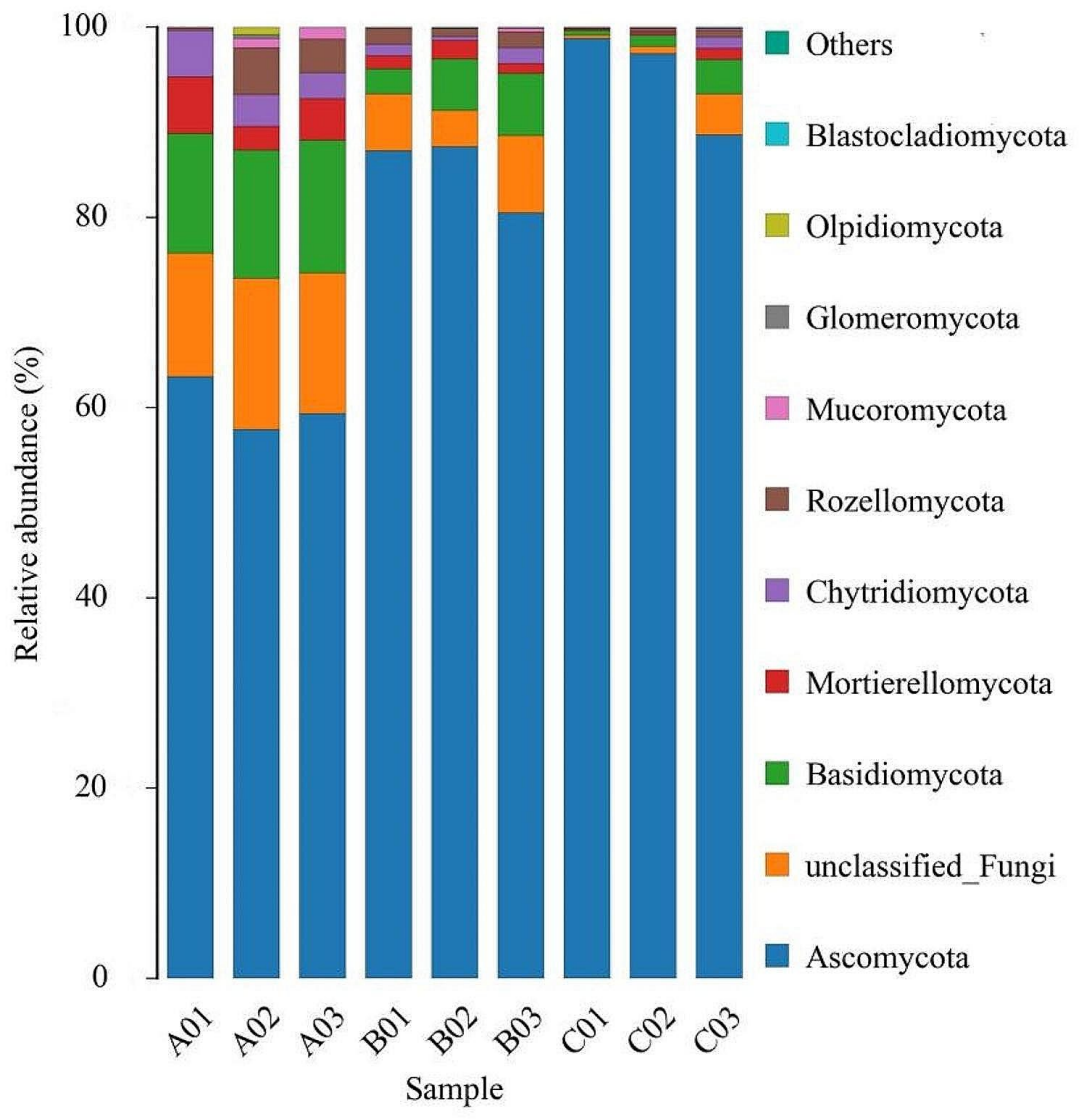
Relative abundance at the phylum level of different groups of *Spatholobus suberectus* Dunn

**Fig. 3:**
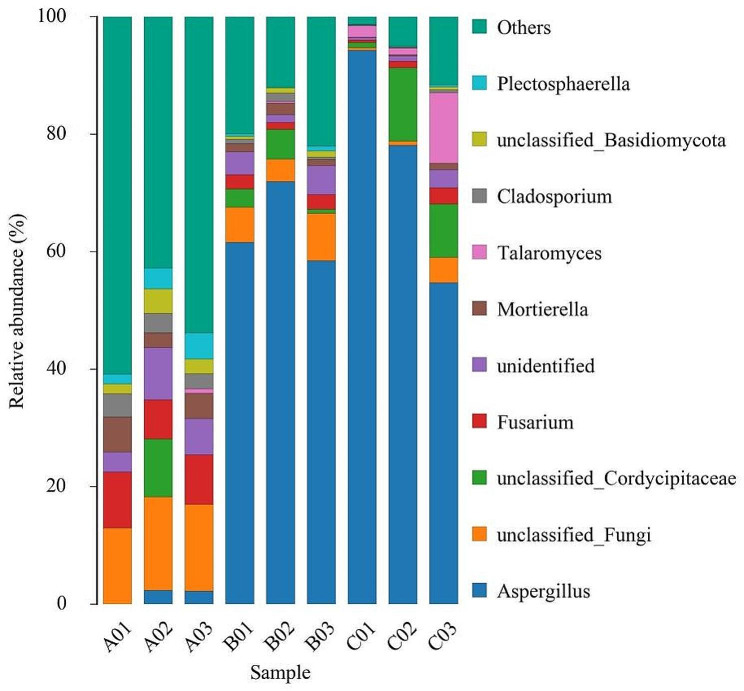
Relative abundance at the genus level of different groups of *Spatholobus suberectus* Dunn

As shown in Figs. 2 and 14 fungal phyla were detected in all samples. The dominant fungi of all samples belonged to *Ascomycota* at the phylum level with the relative abundance of 60.12% in the group A, 85.00% in group B and 94.92% in group C. As shown in Figs. 3 and 336 fungal genera were detected in all samples. The dominant fungus of group A was not obvious, while dominant fungi of group B and group C belonged to *Aspergillus* at the genus level, with relative abundance of 63.99% and 75.72%, respectivel.

### Analysis of *aspergillus* fungi count

**Fig. 4:**
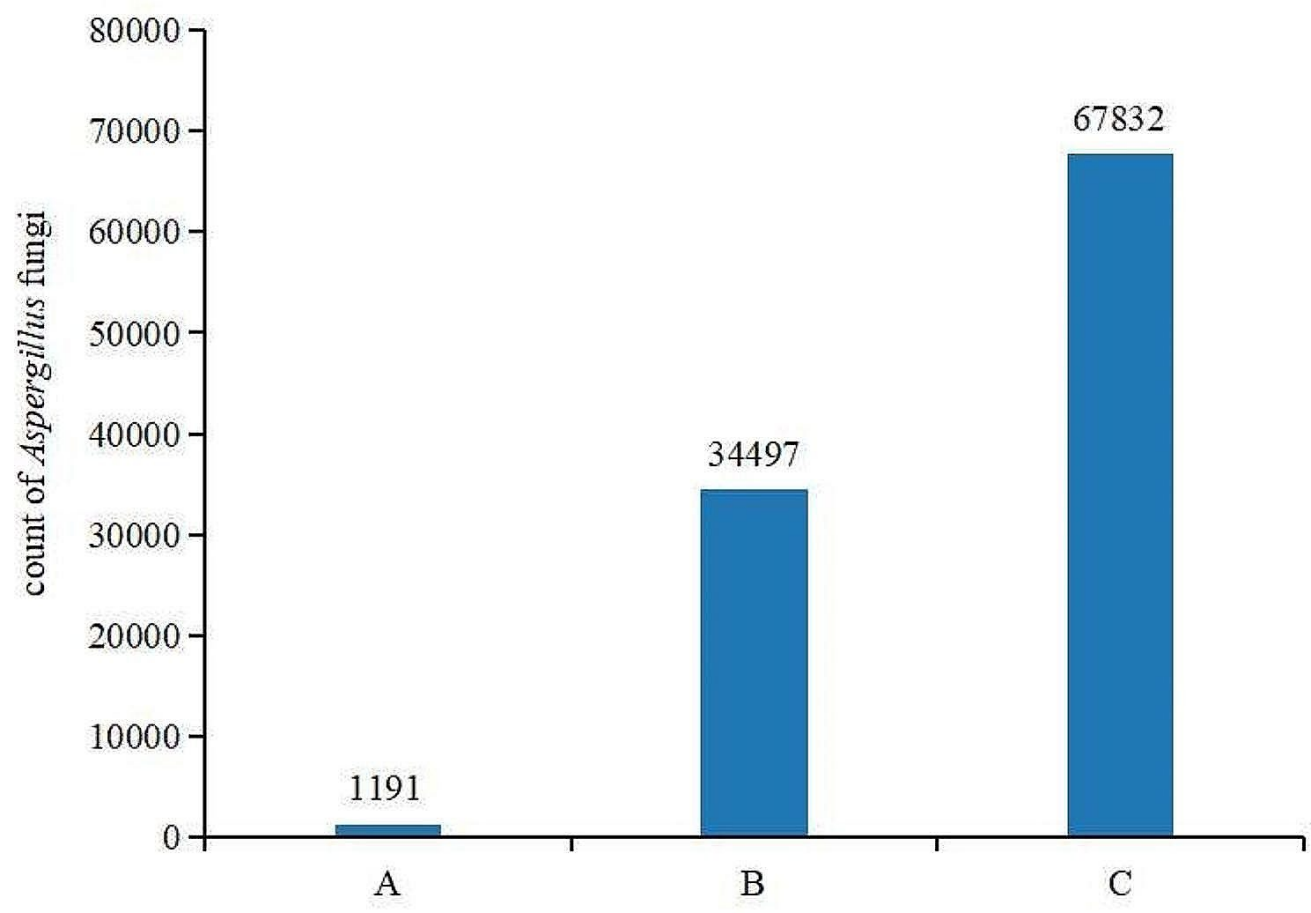
The count of *Aspergillu* fungi of different groups of *Spatholobus suberectus* Dunn

As shown in Fig. 4, the count of *Aspergillus* fungi was 1,191 in group A, 34,497 in group B and 67,832 in group C. The count of *Aspergillus* fungi was in group B was about 28.9 times that in group (A) The count of *Aspergillus* fungi in group C was about 1.9 times that in group (B) It indicated that *Aspergillus* fungi multiplied in large numbers at the middle stage of mildew, and the reproduction rate slowed down with further extension of mildew time.

### α-diversity analysis

α-diversity reflects the species abundance and diversity of a single sample, and there are different indexes to elaborate it. For example, Coverage index was used to estimate the coverage of specie, ACE index and Chao1 index were used to estimate the richness of microorganisms in the sample, and Simpson index and Shannon index were used to estimate the diversity of microbial communities.

As shown in Table 1, the Coverage index of all groups reached 1.00, indicating that the sequencing results could completely reflect the composition of fungal communities of *S*. *suberectus* Dunn samples. Chao1 index and ACE index of group C were higher than those in other groups with significant difference (*P* > 0.05), indicating the abundance of fungal communities of *S*. *suberectus* Dunn samples at the late stage of mildew increased significantly. The Simpson index and Shannon index of the samples in group B and group C were lower than those in group A with significant difference, indicating the diversity of fungal community of *S*. *suberectus* Dunn. samples gradually decreased with the extension of mildew time.

**Table 1:** Fungal community α-diversity index in samples from different groups of *Spatholobus suberectus* Dunn

No.	ACE Index	Chao1 Index	Simpson Index	Shannon Index	Coverage Index
A	177.32 ± 19.04b	176.79 ± 18.59b	0.99 ± 0.00a	6.89 ± 0.15a	1.00
B	150.22 ± 24.29b	150 ± 24.64b	0.69 ± 0.11b	3.81 ± 0.76b	1.00
C	360.14 ± 131.84a	359.69 ± 131.51a	0.74 ± 0.05b	3.19 ± 0.70b	1.00

a, b, c means significant difference at 0.05

### PCoA and NMDS analysis

**Fig. 5:**
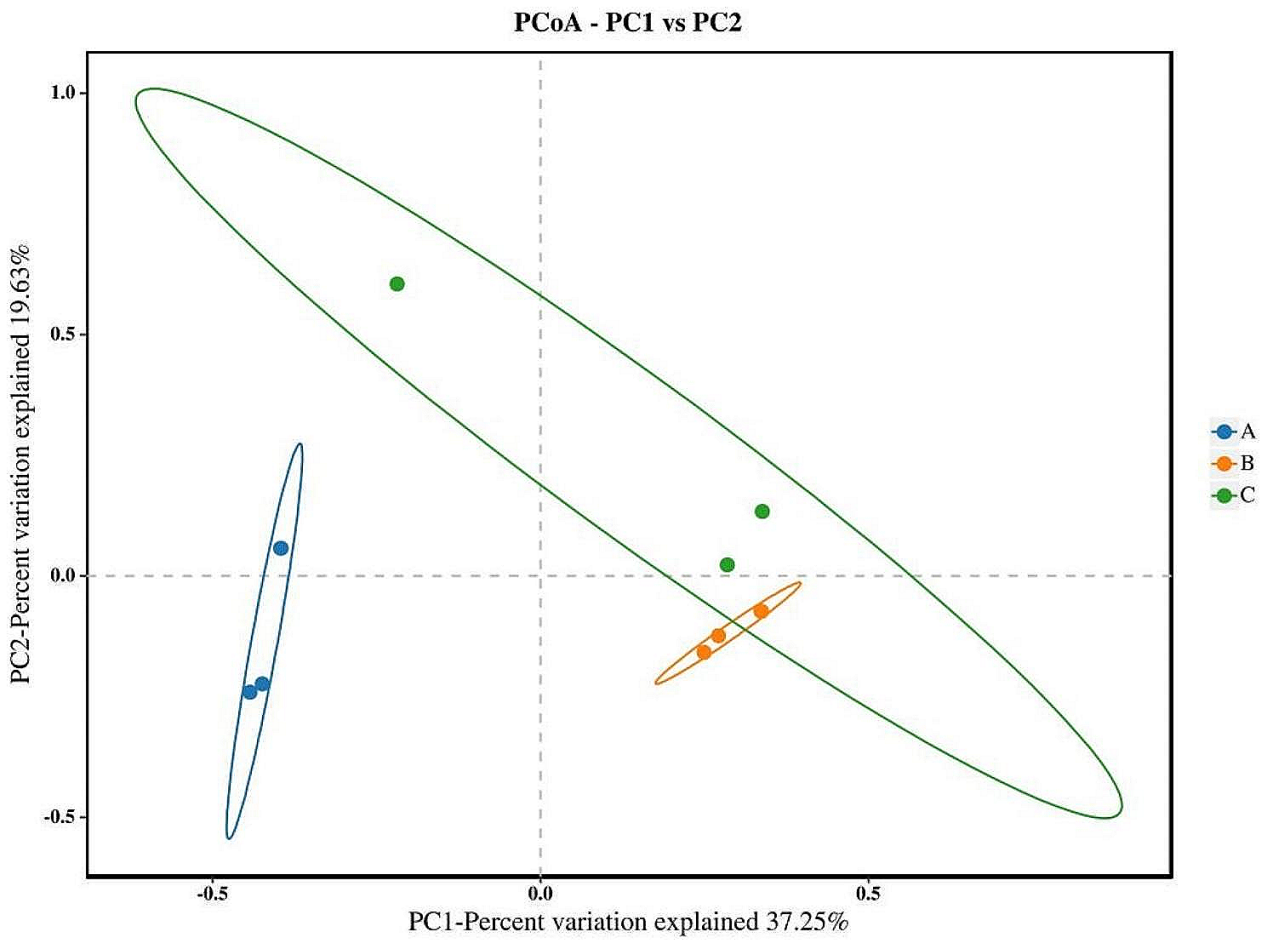
PCoA of microbial flora of different groups of *Spatholobus suberectus* Dunn

PCoA (Gower 1996) is a dimensionality reduction ranking method, which was used to classify multiple samples and show the differences in species diversity among the samples. The closer the distance of different samples, the more similar the structure of species composition. As shown in Fig. 5, the distance between group A and PC2 axis is closer than PC1 axis, indicating the structure of fungal community in the samples is greatly affected by principal component. The distance between group B and PC1 axis is closer than PC2 axis, indicating the structure of fungal community in the samples was greatly affected by principal component PC1. The distance between group C and PC1 axis is closer than PC2 axis, indicating the structure of fungal community in the samples was greatly affected by the principal component PC1. According to the principal coordinate analysis, PC1 and PC2 represented the difference of 37.25% and 19.63% respectively. The distribution of the samples in group B and group C was concentrated, and the distance between group A and other groups was relative longer, indicating the structure of fungal community in the *S*. *suberectus* Dunn samples at the early stage of mildew was greatly different from that at other stages of mildew.

**Fig. 6:**
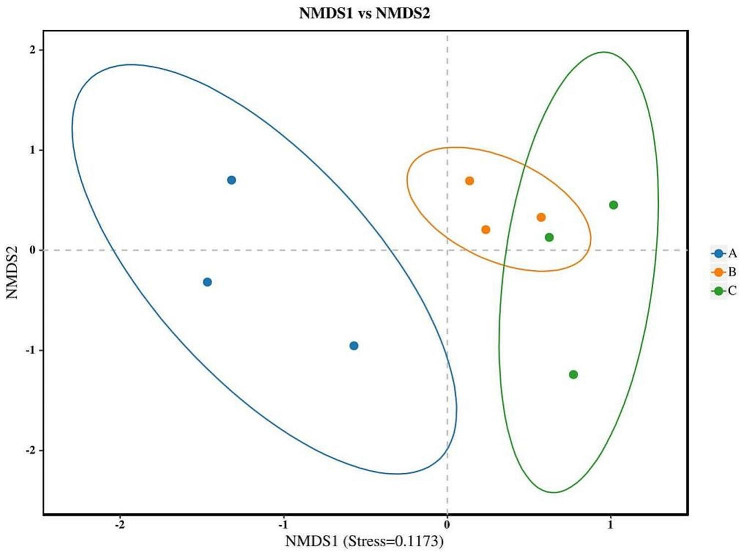
MDS analysis of microbial flora of different groups of *Spatholobus suberectus* Dunn

NMDS (Khardori 2012) is a ranking method, which was applicable for ecological research to simplify research objects from multidimensional space to lower dimensional space for location, analysis and classification, while preserving the original relationships between objects. The degree of difference between different samples is reflected by the distance of different points. The closer the distance is, the more similar the sample composition is. As shown in Fig. 6, the distance between group B and group C was shorter, while the distance between group A and other groups was longer, indicating the structure of fungal community in the *S*. *suberectus* Dunn samples at the early stage of mildew was significantly different from that at other stages of mildew.

### Analysis of AFB_1_ content

**Fig. 7:**
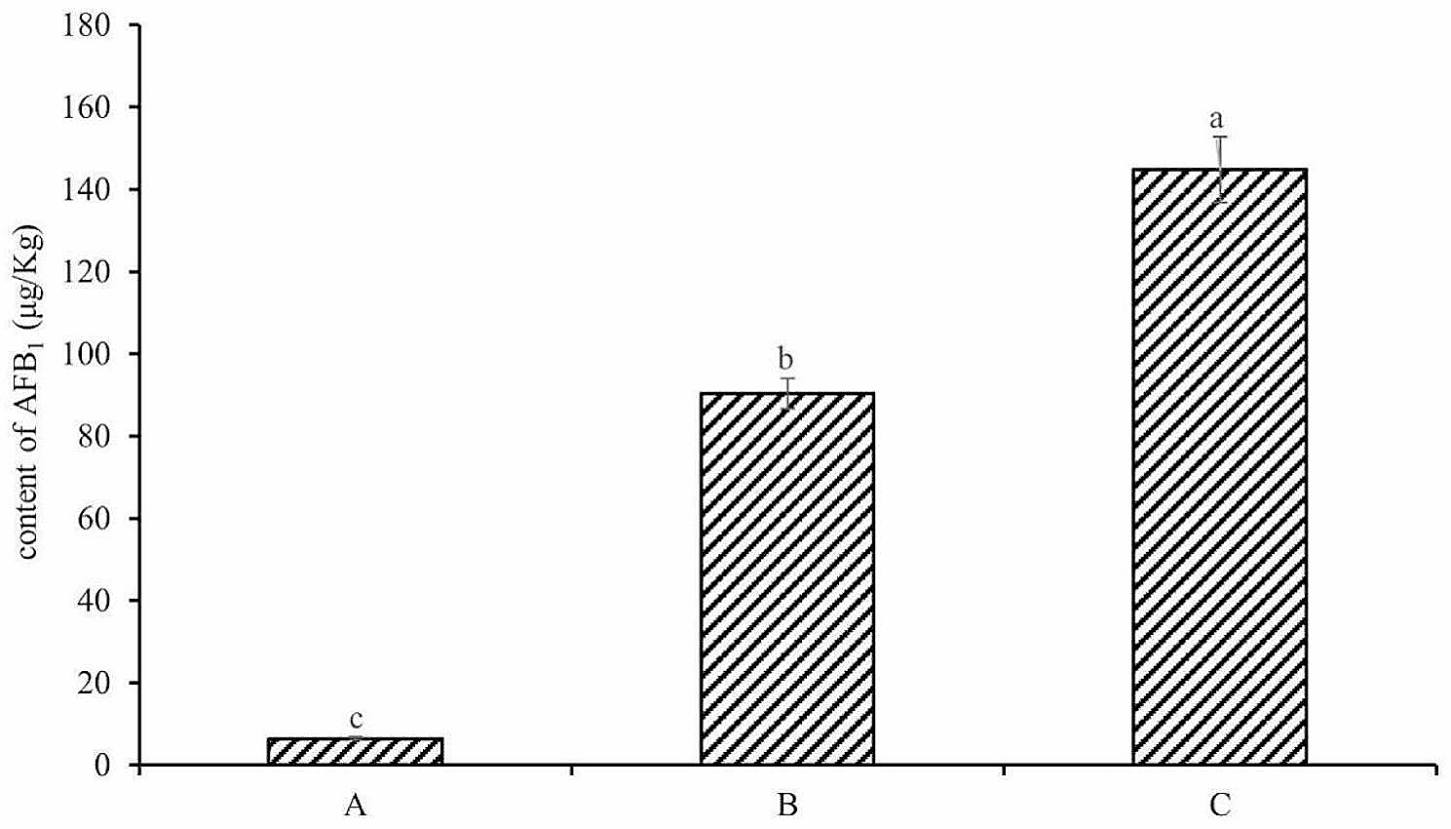
The content of AFB_1_ in different groups of *Spatholobus suberectus* Dunn. a, b, c means significant difference at 0.05

As shown in Fig. 7, the lowest AFB_1_ content was 6.46 µg/Kg in group A. The second highest was 90.33 µg/Kg in group B, which was about 13.9 times that in group A. The highest was 144.79 µg/Kg in group C, which was about 1.6 times of that in group B, and there was a significant difference among all groups (*P* < 0.05). It indicated that the content of AFB_1_ in the *S. suberectus* Dunn samples increased with the extension of mildew time, and inreased rapidly at the middle stage of mildew and then slowed down, which was positively correlated with the increasing trend of *Aspergillus* fungi count. Accoriding to the research, the AFB_1_ is a secondary metabolite of *Aspergillus* fungi (Liu et al. 2021), and AFB_1_ usually produced more at early stages of growth (Xu 2008). Therefore, the reason for above phenomenon may be that the content of AFB_1_ is related to the *Aspergillus* fungi count, but also to the fungi growth stage.

### Analysis of flavonoid content

**Fig. 8:**
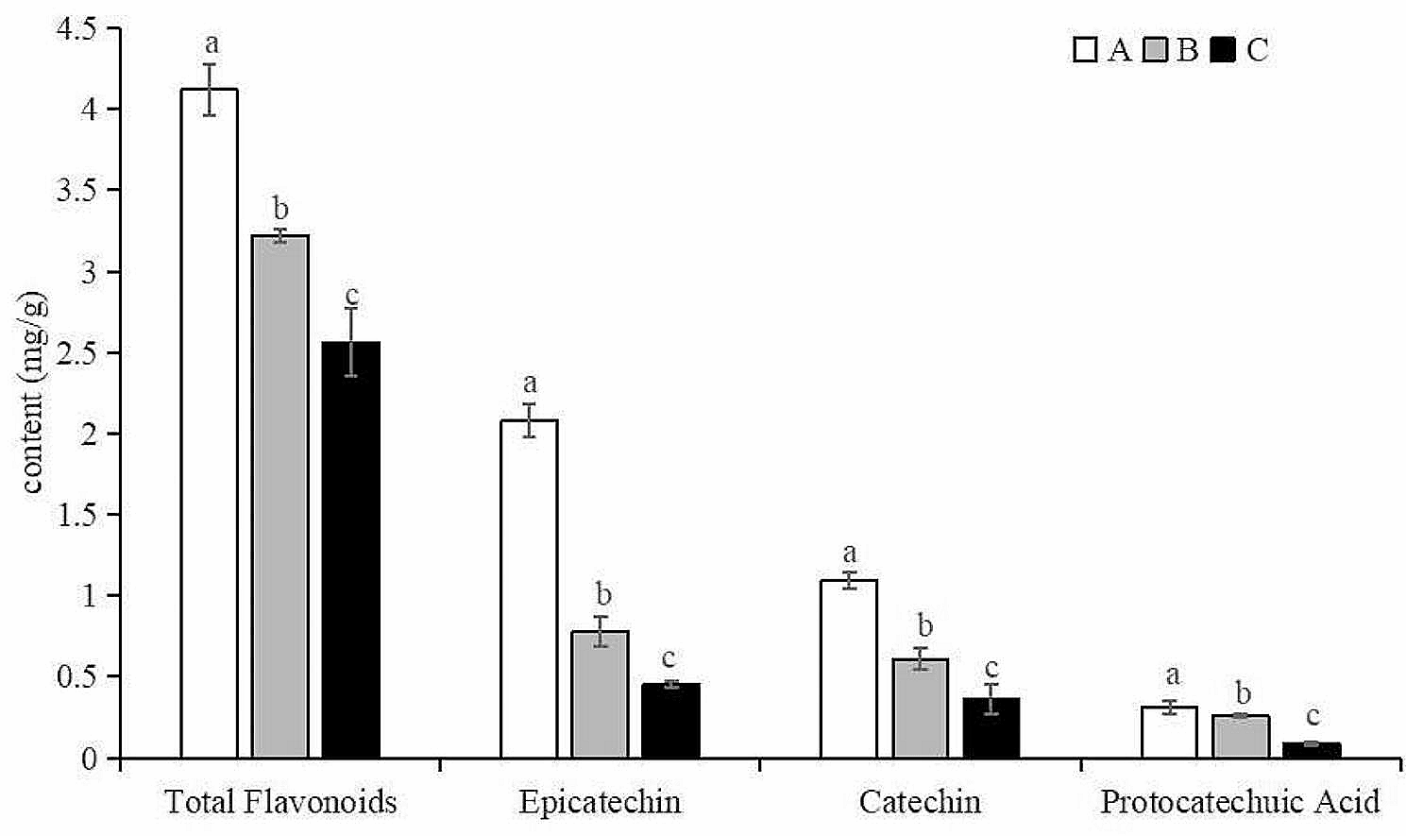
The content of flavonoid components in different groups of *Spatholobus suberectus* Dunn. a, b, c means significant difference at 0.05

As shown in Fig. 8, the contents of total flavonoids in group A, B, C were 12.00, 3.22, 2.56 mg/g, respectively. The contents of epicatechin in group A, B, C were 2.08, 0.78 0.45 mg/g, respectively. The contents of epicatechin in group A, B, C were 1.09, 0.61, 0.36 mg/g, respectively. The contents of protocatechuic acid in group A, B, C were 0.31, 0.26, 0.09 mg/g, respectively. In brief, the content of flavonoids was the highest in group A, followed by those in group B, and the lowest in group C, and there was a significant difference among all groups (*P <* 0.05). The result indicated the content of flavonoids in the *S*. *suberectus* Dunn samples reduced with the extension of mildew time. The reason for this phenomenon may be that the biological abundance of fungi increases with the extension of the mildew period, and fungi have the role of decomposition of active components (Liu, 2015), thus the content of flavonoids decreased accordingly.

## Discussion

As a traditional Chinese herbal medicine, *S. suberectus* Dunn grows in southern regions of China, where climatic conditions are suitable for the growth of mold. Thus, mildew is easily produced during the harvest and storage of *S. suberectus* Dunn, which often leads to the decrease of the active ingredients content. In addition, some molds can produce mycotoxins that threaten human health, the most common being AFs, which has been listed the most dangerous food hazard in nature by the Food and Agriculture Organization (FAO) of the United Nations and the World Health Organization (WHO).

High-throughput sequencing technology is a new generation of molecular biology technology developed at the beginning of 21 century, which has the advantages of accuracy, flexibility, high throughput and low cost (Hall 2007). Recently, it has become a hot spot in the study of microbial diversity of Chinese herbal medicine, such as *Lycii Fructus* and *Platycladi Semen* (Yu 2022). In this paper, the Chao1, ACE, Simpson and Shannon indexes comprehensively reflected the fungal community richness and diversity of *S*. *suberectus* Dunn in different mildew periods. The richness indexes showed that the fungal community richness of samples at the early stage of mildew was the least, followed by that at the middle stage of period, and that at the late stage of mildew was the largest. The diversity indexes showed that the fungal community diversity of samples was the largest at the early stage of mildew, followed by that at the middle stage of mildew, and that at the late stage of mildew was the least. The results of species community composition and structure analysis showed that, at the phylum level, the dominant fungi in all stages of mildew belonged to *Ascomycota.* At the genus level, the dominant fungi at the early stage of mildew was not obvious, whereas the dominant fungus at the middle and late stages of mildew belonged to *Aspergillus*, and its count increased with the extension of mildew time. The results of PCoA and NMDS analysis showed that the structure of fungal community in the samples at the early stage of mildew was different from that in the samples at other stages of mildew. The reason for the above phenomenon may be that with the extension of the mildew time, more and more fungi began to reproduce, so the number of species gradually increased, meaning the species richness gradually increased. At the same time, due to the growth competition between different species, the growth advantage of *Aspergillus* gradually became prominent, resulting in the uneven distribution of the individual number of different species, meaning the species diversity reduced, and the structure of fungal community changed accordingly.

Mildew has a great impact on the quality of Chinese herbal medicine, not only reducing the content of active ingredients in herbs, but also accumulating mycotoxins. In this paper, flavonoids and AFB_1_ were used as indicators of active ingredients and mycotoxins in the samples of *S. suberectus* Dunn and their contents were monitored. The results showed that the content of flavonoids gradually decreased with the extension of mildew time, while the content of AFB_1_ gradually increased, which was similar to the results studied by Zhao et al. about the quality evaluation of *Alpinia oxyphylla* after *Aspergillus* flavus infection (Zhao et al. 2017).

Chinese herbal medicine is a valuable resource for traditional medicine in China. Moreover, it is the basis for the survival and sustainable development of Chinese medicine industry. Therefore, it is necessary to adopt suitable conservation methods for Chinese herbal medicines to minimize the rate of mildew during harvest and storage. This paper will provide a theoretical basis for the development of new anti-molding technology.

## Data Availability

All sequnce data were submitted to NCBI, Accession to cite for these SRA data: PRJNA1016145.

## Abbreviations

*S. suberectus* Dunn: *Spatholobus suberectus* Dunn
HPLC: high-performance liquid chromatography
UV-spectrophotometry: ultraviolet spectrophotometry
ELISA: enzyme-linked immunosorbent assay
AFB_1_: Aflatoxins B_1_
AFs: aflatoxins
OTU: operational taxonomic unit
PCoA: Principal ordinates analysis
NMDS: Non-MetricMulti-Dimensional Scaling
FAO: Food and Agriculture Organization
WHO: World Health Organization
IARC: International Agency for Research on Cancer
